# Fetal Congenital Heart Block Associated With Maternal Primary Systemic Lupus Erythematosus and Sjogren’s Syndrome

**DOI:** 10.7759/cureus.18036

**Published:** 2021-09-16

**Authors:** Sameera Khan, Priyanka Anvekar, Petras Lohana, Mohammed Sheeraz Alam, Syed R Ali

**Affiliations:** 1 Internal Medicine, Lala Lajpat Rai Memorial Medical College, Aligarh, IND; 2 Medicine and Surgery, Mahatma Gandhi Missions (MGM) Medical College and Hospital, Mumbai, IND; 3 Internal Medicine, Liaquat University of Medical and Health Sciences Hospital, Karachi, PAK; 4 Internal Medicine, Jawaharlal Nehru Medical College, Aligarh, IND; 5 Internal Medicine, Dow University of Health Sciences, Civil Hospital Karachi, Karachi, PAK

**Keywords:** congenital heart block, systemic lupus erythromatosus, internal medicine and rheumatology, cardiac pacemaker, sjogren's disease

## Abstract

Congenital heart block is a grave condition reported in 0.5% of 100 live births. Systemic lupus erythematosus (SLE) and Sjogren's syndrome (SS) are chronic autoimmune and inflammatory condition, which affects multiple systems. The association of SLE and SS with pregnancy has been seen in the past. Usually, it shows anti-Ro/SSA and anti-Ro/SSB auto-antibodies in maternal serum, which is proportional to fetal Outcome. In this report, we present a case of a 29-year-old female gravida 4, para one and aborta 3, with a history of polycystic ovarian disease and multiple abortions. At 20 weeks of gestation, her antenatal examination revealed fetal bradycardia and heart block, which further led to SLE and SS diagnosis in her. She was treated with steroids to prevent further fetal complications. The patient delivered a healthy neonate at 38 weeks of gestation. The neonate eventually received a cardiac pacemaker and is now on regular follow-up.

## Introduction

Systemic lupus erythematosus (SLE) and Sjogren's syndrome (SS) are autoimmune diseases that exhibit the features of long-standing, persistent inflammatory changes affecting multiple systems with a preference for young women [1]. SLE and SS display many features that overshadow each other and have been associated with grave side-effects during pregnancy [2,3]. Fetal heart block is the most common manifestation in pregnancy, presenting either as a conduction defect or an isolated abnormality [4]. We report a case of a 29-year-old female, gravida four and para 1, presenting at 20 weeks of gestation and with a history of multiple abortions and polycystic ovarian disease for a routine antenatal checkup. Detailed evaluation leads to the findings of complete heart block in the fetus and diagnosis of SLE with concomitant SS in the mother. We also highlight and discuss the importance of timely diagnosis of this notorious disease and briefly review the management.

## Case presentation

A 29-year-old female G4P1 presented at 20 weeks of gestation in the outpatient department for an antenatal checkup. She has a history of multiple abortions and polycystic ovarian disease. She has a healthy four-year-old daughter; her last miscarriage was a year back at eight weeks of pregnancy. Since then, her fasting insulin was very high, and she was started on metformin 500mg BID. She was taking treatment from the infertility clinic due to multiple abortions. On physical examination: she was vitally stable, blood pressure 90/60 mmHg, mean arterial pressure 70 mmHg, weight 65 kg, pulse 75 beats/min, BMI 24.5, SpO_2_ 98%, temperature 36.8 ºC. There was no swelling of the hand and feet, fundal height at the umbilicus, but missed heartbeats on fetal examination. The patient was advised for fetal ultrasound, which revealed missed fetal heartbeat in a real-time scan. A referral was given to the cardiologist. Echo showed persistent bradycardia over the whole examination period with a fetal heart rate of 65 beats/min (Video 1).

**Video 1:** Echo showing persistent bradycardia

There were no signs of fetal heart decompensation at this stage. The cardiologist advised for auto-antibody profile, rubella antibody, and thyroid profile, and she was given Dexamethasone 6mg two times per day for two days to prevent further complications in the fetus. The laboratory reports are listed in Table 1.

**Table 1:** The laboratory investigations after initial routine blood investigations. * Second Trimester

Investigation	Result	Normal reference value
TSH, by ECLIA (µIU/mL)	5.15	0.2-3.0*
Rubella- IgG, serum (IU/mL)	23.0	More than 10.0
Anti-Sm	14	0-5.0
Anti SS-A (Ro)	58	0-5.0
Anti Ro-52	120	0-5.0
Anti SS-B (La)	101	0-5.0
Anti-thyroglobulin (ATG) (IU/mL)	22.66	Less than 4.11

Based on the laboratory results SLE with SS was diagnosed. She was started on aspirin 100mg, clexane 40mg daily and advised to follow up with a rheumatologist for her recent diagnosis of SLE and SS. She delivered a full-term fetus via normal delivery at 38 weeks of gestation. The neonate was healthy without any cardiac abnormality, except there was bradycardia which was consistent. The Apgar score at one and five minutes was 1 and 6, respectively. A pediatric cardiology consult was given, and it was decided to insert a temporary pacemaker. On day 4, a permanent pacemaker was placed. The neonate continued care under the neonatologist and pediatric cardiologist after discharge.

## Discussion

SLE is a long-standing autoimmune disorder that can affect any part of the human body leading to inflammatory changes. It mainly affects young women, and the manifestations are known to worsen in pregnancy due to high levels of estrogen [5]. SS is another autoimmune disease associated with severe outcomes during pregnancy [6]. However, the current literature rarely mentions the simultaneous presence of SLE and SS during pregnancy inducing complete heart block in the fetus as a pregnancy-related complication [7]. Fetal congenital heart block (CHB) is the most common outcome of pregnancies related to SLE and SS. It is seen in approximately 0.5 women testing positive for anti-SS-A/Ro and/or anti-SS-B/La antibodies, and the incidence increases with consequent pregnancies [7]. Moreover, the presence of antinuclear antibodies (ANAs) is considered a hallmark in marking SLE diagnosis [8]. Our patient has a remarkable history of multiple abortions in the past and was consequently diagnosed with high levels of anti-SS-A and anti-SS-B antibodies in the current pregnancy, confirming the diagnosis of SLE and SS. The autoantibodies produced by the maternal body transplacentally enters the fetal circulation, which leads to conduction defects by involving the atrioventricular tissues of the fetal heart due to inflammatory changes or by interfering with the ion channel leading to fibrosis [9-11]. Fetal echocardiography is the quickest and easiest way to diagnose fetal CHB. Regular antenatal checkups and assessing the mothers with fetal echocardiography early in the pregnancy can help identify the ongoing conduction defects, fetal discomfort as well as hydrops fetalis [12]. The antenatal visit at 20 weeks in our patient revealed fetal bradycardia that prompted immediate assessment with fetal echocardiography confirming the diagnosis of CHB in the fetus. The management mainly comprises organized and timely monitoring for heart block and treating the first known heart block with fluorinated steroids given transplacentally. Prophylactic treatment with steroids is not recommended due to maternal and fetal after-effects [13,14]. Almost all the patients require a permanent pacemaker in the later years of childhood, and this has shown favorable outcomes in terms of mortality and morbidity [15]. Our patient responded well to the steroid therapy and delivered a healthy child generally at term. The patient is now on regular follow-up with her rheumatologist and pediatrician for neonatal care.

## Conclusions

In conclusion, pregnancy associated with SLE and SS is rare and exhibits pregnancy-related severe complications. Fetal CHB is one of the most commonly encountered outcomes and is diagnosed with the help of a fetal echocardiogram. The mothers are managed with transplacental steroids. However, the children eventually require a permanent pacemaker. We intend to report this uncommon case to add more knowledge on this topic in the literature.
